# Temporal X-ray Reconstruction using Temporal and Spectral Measurements at LCLS

**DOI:** 10.1038/s41598-020-66220-5

**Published:** 2020-06-17

**Authors:** Florian Christie, Alberto Andrea Lutman, Yuantao Ding, Zhirong Huang, Vatsal A. Jhalani, Jacek Krzywinski, Timothy J. Maxwell, Daniel Ratner, Juliane Rönsch-Schulenburg, Mathias Vogt

**Affiliations:** 1 Deutsches Elektronen-Synchrotron, 22607 Hamburg, Germany; 2 Universität Hamburg, 22761 Hamburg, Germany; 3 SLAC National Accelerator Laboratory, Menlo Park, California, 94025 USA; 4 Present Address: California Institute of Technology, Pasadena, California 91125 USA

**Keywords:** X-rays, Ultrafast photonics, Free-electron lasers

## Abstract

Transverse deflecting structures (TDSs) are widely used in accelerator physics to measure the longitudinal density of particle bunches. When used in combination with a dispersive section, the whole longitudinal phase space density can be imaged. At the Linac Coherent Light Source (LCLS), the installation of such a device downstream of the undulators enables the reconstruction of the X-ray temporal intensity profile by comparing longitudinal phase space distributions with lasing on and lasing off. However, the resolution of this TDS is limited to around 1 fs rms (root mean square), and therefore, it is not possible to resolve single self-amplified spontaneous emission (SASE) spikes within an X-ray photon pulse. By combining the power spectrum from a high resolution photon spectrometer and the temporal structure from the TDS, the overall resolution is enhanced, thus allowing the observation of temporal, single SASE spikes. The combined data from the spectrometer and the TDS is analysed using an iterative algorithm to obtain the actual intensity profile. In this paper, we present some improvements to the reconstruction algorithm as well as real data taken at LCLS.

## Introduction

Transverse deflecting structures (TDSs) are used to time-resolve the electron bunch phase spaces downstream of an X-ray free-electron laser (FEL) undulator line to measure the electron bunch energy losses induced by the lasing process^1^. The electron bunch time-resolved losses match the emitted X-ray temporal profile^2^, and therefore TDSs are routinely used as diagnostics to measure the X-ray pulse profiles^1^. However, the limited resolution of a measurement using a TDS imposes an upper limit on the resolution of the temporal reconstruction of photon pulses. As the resolution of an X-band TDS used at an X-ray FEL is typically limited to around 1 fs rms (root mean square), single self-amplified spontaneous emission (SASE) spikes, typically in the range of 0.1 fs to 3 fs, within one photon pulse can often not be resolved. However, the exact knowledge of the temporal structure of SASE radiation is interesting for applications such as “ghost imaging”^3^.

By combining the power spectrum from a high resolution photon spectrometer^4^ and the temporal structure from the TDS, the overall resolution can be enhanced, thus allowing the observation of temporal, single SASE spikes in the X-ray range. The combined data from the spectrometer and the TDS is analysed using an iterative algorithm to obtain an estimate of the actual intensity profile. This iterative reconstruction algorithm is published in previous work^5,6^.

**Figure 1:**
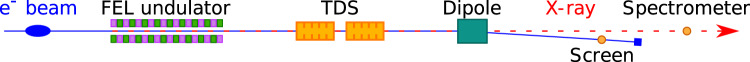
Schematic of the measurement layout. An electron beam is sent through an FEL undulator where it produces X-ray radiation. Downstream of the FEL undulator, a horizontally deflecting TDS maps the longitudinal position to the horizontal. Additionally, a vertically deflecting dipole is used to separate the X-ray photons and the electrons and to relate the vertical bunch coordinate to the energy of the electrons. The longitudinal phase space density can then be measured using a screen. From these measurements it is possible to determine the temporal X-ray pulse profile. The X-rays are sent through a high-resolution spectrometer, where the power spectrum can be measured. Both measurements are combined for the temporal reconstruction of a photon pulse.

A schematic of the measurement layout at the Linac Coherent Light Source (LCLS) is shown in Fig. 1.

In the following, we will discuss some improvements and adjustments to this iterative reconstruction algorithm that are necessary to analyse real data due to the spectrometer resolution of 0.2 eV during the measurements.

## Adjustments to iterative reconstruction algorithm

The iterative reconstruction algorithm is described in detail in previous work^5,6^. The blurred temporal profile $\tilde{P}(t)$ measured by a TDS and the blurred power spectrum $\tilde{{\mathscr{P}}}(\omega )$ measured by a spectrometer are the measured data used by the reconstruction algorithm to retrieve the actual pulse profile. The mechanism by which the finite TDS resolution blurs the actual temporal intensity profile *P*(*t*) is modelled by a convolution with a Gaussian *G*(*t*) of fixed standard deviation *R*_*t*_

1 $$(P\ast G)(t)=\tilde{P}(t).$$

In contrary to the first publication of the algorithm^5^, in this paper we assume that also the power spectrum ${\mathscr{P}}(\omega )$ measured by the spectrometer has a limited resolution *R*_*ω*_. This process is also modelled by a convolution with a Gaussian *G*(*ω*) of width *R*_*ω*_

2 $$({\mathscr{P}}\ast G)(\omega )=\tilde{{\mathscr{P}}}(\omega \mathrm{)}.$$

The blurred temporal profile $\tilde{P}(t)$ and the blurred power spectrum $\mathop{{\mathscr{P}}}\limits^{ \sim }(\omega )$ are the starting points for the algorithm.

### Linearly chirped base functions

The electric field of the photon pulse to be approximated is modelled as a sum of in principle arbitrary base functions *B*_*j*_(*t*) in time with varying complex coefficients *a*_*j*,*m*_, where *m* is the iteration step,

3 $${F}_{m}(t)=\mathop{\sum }\limits_{j=1}^{n}\,{a}_{j,m}{B}_{j}(t)$$

as is the field in the frequency domain

4 $${{\mathscr{F}}}_{m}(\omega )=\mathop{\sum }\limits_{j=1}^{n}\,{a}_{j,m}{{\mathscr{B}}}_{j}(\omega ),$$

where ${{\mathscr{B}}}_{j}(\omega )$ are the base functions in the frequency domain. We note here, that the base functions are chosen in a range, where we expect the temporal and the spectral distributions of the photon pulse to be non-zero due to prior information on the photon emission process from FEL theory^7,8^.

The Gaussian base functions described in the previous work^5^ are not chirped in time as suggested by the literature^9–11^. To accommodate this we introduce an arbitrary linear chirp factor *β*_*j*_ to the base functions

5 $${B}_{j}(t)={\left(\frac{1}{\sqrt{2\pi }{\sigma }_{j}}\right)}^{\frac{1}{2}}{e}^{-\frac{{(t-{t}_{j})}^{2}}{4{\sigma }_{j}^{2}}}{e}^{i{\omega }_{j}t}{e}^{i{(t-{t}_{j})}^{2}{\beta }_{j}},$$

where *σ*_*j*_ is the width of the Gaussians centred at times *t*_*j*_, and the *ω*_*j*_ can be initially calculated based on the energy profile of the electron phase space. For a linearly chirped electron bunch, we can for example set ${\omega }_{j}={\omega }_{0}+2\frac{{\gamma }_{j}-{\gamma }_{0}}{{\gamma }_{0}}{\omega }_{0}$^12^, where *ω*_0_ is the main radiation frequency created by electrons with an energy of *γ*_0_ and *γ*_*j*_ is the mean energy of the electrons around *t*_*j*_. Otherwise, they are initialized as *ω*_*j*_ = *ω*_0_.

These base functions are chosen in a way that

6 $$\begin{array}{rcl}{\int }_{-\infty }^{\infty }\,{|{B}_{j}(t)|}^{2}\,{\rm{d}}t & = & {\int }_{-\infty }^{\infty }\,\frac{1}{\sqrt{2\pi }{\sigma }_{j}}\cdot {e}^{-\frac{{(t-{t}_{j})}^{2}}{2{\sigma }_{j}^{2}}}\,{\rm{d}}t\\  & = & 1.\end{array}$$

By setting ${C}_{j}=\frac{1}{4{\sigma }_{j}^{2}}-i{\beta }_{j}$, Eq. (5) becomes

7 $${B}_{j}(t)={\left(\frac{1}{\sqrt{2\pi }{\sigma }_{j}}\right)}^{\frac{1}{2}}{e}^{-{(t-{t}_{j})}^{2}{C}_{j}}{e}^{i{\omega }_{j}t}$$

and we obtain the base functions in frequency domain

8 $${{\mathscr{B}}}_{j}(\omega )={\left(\frac{1}{\sqrt{2\pi }{\sigma }_{j}}\right)}^{\frac{1}{2}}\frac{1}{\sqrt{2{C}_{j}}}{e}^{-\frac{(\omega -{\omega }_{j})(-4i{C}_{j}{t}_{j}+\omega +{\omega }_{j})}{4{C}_{j}}}.$$

Following the literature^10,11^ we set ${\beta }_{j}=-\frac{1}{4\sqrt{3}{\sigma }_{j}^{2}}$ for all base functions.

The iteration process and the minimizing is described in detail in the previous work^5^. The only change applied to the algorithm is that instead of the actual power spectrum ${\mathscr{P}}(\omega )$ the blurred power spectrum $\tilde{{\mathscr{P}}}(\omega )$ is used for the projected spectrum for the real data taken at LCLS.

### Testing of the algorithm using a blurred power spectrum

Similar to the prior publication^5^ the algorithm is tested using simulation data of LCLS at 1.5 nm with a blurred power spectrum using a resolution of *R*_*ω*_ = 0.2 eV rms. This value was chosen to match the resolution of the spectrometer used for measurements at LCLS. Therefore, the same tests as in the previous work^5^ are conducted and showcased in the following using a blurred power spectrum $({\mathscr{P}}\ast G)(\omega )=\tilde{{\mathscr{P}}}(\omega )$ with *R*_*ω*_ = 0.2 eV. FEL simulations^10^ for bunch charges of 20 pC, 40 pC, and 150 pC are again used for the testing.

**Figure 2:**
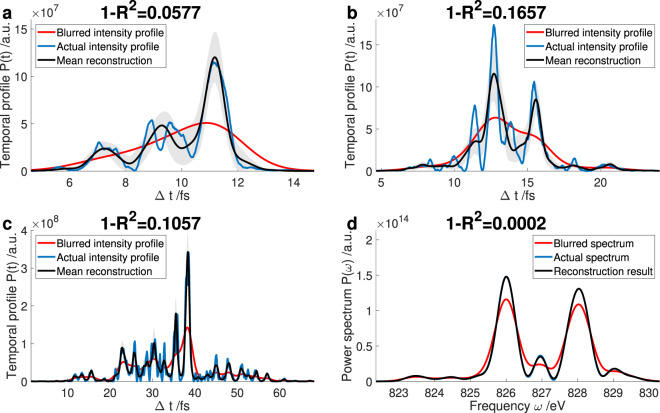
Reconstruction results for simulations of the FEL process. The temporal resolution is *R*_*t*_ = 1 fs. Temporal profiles are shown for FEL pulses from bunches with a charge of 20 pC (**a**), 40 pC (**b**), and 150 pC (**c**). A reconstructed power spectrum for the temporal profile of (**a**) is shown in (**d**). The spectral resolution is *R*_*ω*_ = 0.2 eV.

Every reconstruction result is obtained by averaging 50 individual reconstructions, where different random coefficients *a*_*j*,0_ are used to construct the initial base functions. Individual results for bunch charges of 20 pC to 150 pC are shown in Fig. 2. The actual intensity profile *P*(*t*) is plotted using a blue line, the blurred intensity profile $\tilde{P}(t)$ is plotted using a red line. The TDS resolution is *R*_*t*_ = 1 fs in these figures. The reconstruction result $\bar{P}(t)$, which is the mean of the 50 individual reconstructions, is plotted using a black line surrounded by a light gray shaded area that is one standard deviation. The error shown here is the error stemming from the statistics in the inversion process. Not all the reconstructions arrive at the same results for the individual *a*_*j*,*m*_, since multiple temporal and spectral profiles may lead to the same blurred profile^13^. Nonetheless, to obtain a measure for the goodness of the reconstruction the 1 − *R*^2^ value known from statistics is used^5,14,15^

9 $$1-{R}^{2}=\frac{\mathop{\sum }\limits_{j=1}^{n}\,{(P({t}_{j})-\bar{P}({t}_{j}))}^{2}}{\mathop{\sum }\limits_{j=1}^{n}\,{(P({t}_{j})-\langle P\rangle )}^{2}},$$

where $\langle P\rangle$ is the mean value of *P*(*t*). This value can of course only be given for simulations as the exact correct result is then known. If the agreement between the reconstruction result $\bar{P}(t)$ and the actual intensity profile *P*(*t*) is perfect, the 1 − *R*^2^ value is zero. As can be observed, the reconstruction algorithm is still able to retrieve the actual intensity profile very similar to the cases without spectral blurring shown in previous work^5^. The 1 − *R*^2^ values are all in the same region as in^5^ and also the capabilities and limitations of the algorithm remain the same as before. We therefore conclude that even though an uncertainty due to signal processing remains^13^, the algorithm enables an improvement of the temporal X-ray reconstruction of FEL pulses.

Additionally to the temporal profiles, the result of the spectrum reconstruction for the 20 pC bunch is displayed in Fig. 2d. In this case, the actual power spectrum ${\mathscr{P}}(\omega )$ is plotted using a blue line, the blurred power spectrum $\tilde{{\mathscr{P}}}(\omega )$ is plotted using a red line. The spectrometer resolution is *R*_*ω*_ = 0.2 eV in these figures. The reconstruction result $\bar{{\mathscr{P}}}(\omega )$, which is the mean of the 50 individual reconstructions, is plotted using a black line surrounded by a light gray shaded area that is one standard deviation. Nearly all reconstructions arrive at the same power spectrum, therefore the black and the blue line coincide and the light gray shaded area is hardly visible.

**Figure 3:**
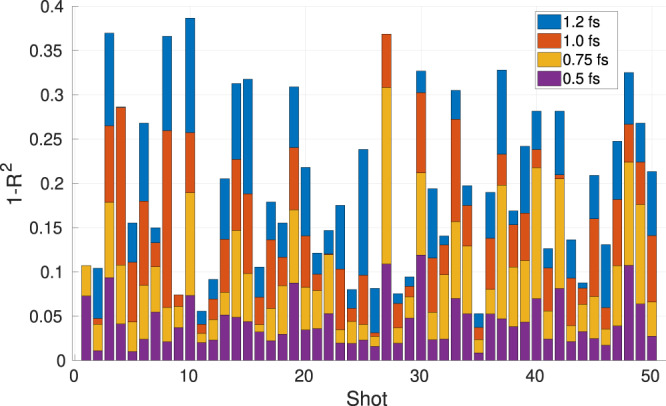
Results of the reconstruction of 50 different SASE shots for 40 pC bunches for different TDS resolutions shown in the legend. The 1 − *R*^2^ value is plotted against the shot number. The power spectrum is blurred using a Gaussian blurring of *R*_*ω*_ = 0.2 eV.

Figure 3 shows the 1 − *R*^2^ values for the 50 different shots with a charge of 40 pC. Comparing to the prior publication^5^ it can be seen, that for every shot the 1 − *R*^2^ value is in the same order for the perfect and the blurred spectrum, respectively. It can therefore be concluded, that the algorithm performs equally well for both spectral measurements.

In summary, the testing results show that the algorithm excels at reconstructing single, isolated spikes and struggles to resolve multiple, dense spikes individually. The existence of adjoining spikes of similar height is retrieved, although the peak power cannot always be correctly determined.

## Iterative reconstruction algorithm applied to measurement data taken at LCLS

A dedicated machine development shift recorded data to be analysed using the iterative reconstruction.

The measurements were taken at a beam energy of 4 GeV resulting in a wavelength of ~1.7 nm or a photon energy of ~730 eV. The initial charge at the cathode was 40 pC, which is later collimated to either 20 pC or 30 pC prior to the undulator. Using energy collimators in the first bunch compressors, electrons with high energy deviations from the reference energy were truncated^16^. An overview of the parameters for the measurements shown in the following can be found in Table 1.

**Table 1:** Parameter Overview of Measurements for Iterative Reconstruction Algorithm at LCLS.

Charge at undulator	30 pC	20 pC
Bunch length *σ*_*t*_	5 fs	3 fs
Peak current *I*_*e*_	2.2 kA	2.5 kA
TDS deflecting voltage *V*	80 MV	80 MV
Temporal resolution *R*_*t*_	1.2 fs	1.0 fs
Spectral resolution *R*_*ω*_	0.2 eV	0.2 eV

**Figure 4:**
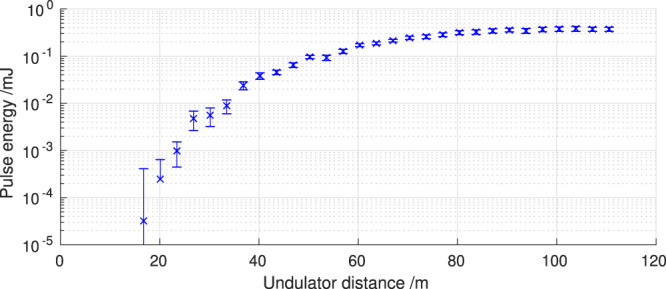
Gain curve measured at LCLS in the course of recording data for the reconstruction algorithm showing the gas detector signal over the distance traveled in the undulators. The error bars indicate one standard deviation. The beam was kicked behind the 20^th^ undulator (at *z* = 67 m) to sustain the unsaturated condition of the bunches, but still provided sufficient signal for the spectrometer.

As the reconstruction has to be performed using bunches that are not yet saturated or close to the saturation point^17^, a gain curve was recorded, see Fig. 4. For this reason the beam was kicked behind the 20^th^ undulator (at *z* = 67 m) to suppress lasing in the downstream undulators. The orbit kick both disrupts overlap between the x-ray waves and electron bunch and degrades electron bunching, interrupting the lasing process^18^. The chosen configuration provided sufficient signal for the spectrometer to work and fulfilled the requirement of being close to the saturation point so that a meaningful reconstruction of the photon pulse power from the energy loss and the energy spread increase of the electron bunch can be performed^17^. The orbit downstream of the undulator section was restored stable by using a closed three-bump^18–20^.

To obtain the photon pulse power the longitudinal phase space density of an electron bunch producing light (lasing on) has to be compared to one where the lasing process is suppressed (lasing off). The longitudinal phase space densities are then divided into slices along the time dimension to get the time-dependent beam parameters such as the mean energy *E*_on,off_(*t*_*i*_), the energy spread ${\sigma }_{{E}_{{\rm{on}},{\rm{off}}}}({t}_{i})$, and the current *I*(*t*_*i*_) in each time slice *t*_*i*_^1,17^. The subscript denotes, whether the quantity was taken from a measurement with lasing on or lasing off. The influence of the FEL process on the bunch current is negligible, therefore, this quantity does not have a subscript. For each time slice, the energy loss and the energy spread increase comparing the lasing-on and lasing-off measurement is then calculated

10 $$\Delta E({t}_{i})={E}_{{\rm{on}}}({t}_{i})-{E}_{{\rm{off}}}({t}_{i}),$$

11 $${\sigma }_{E}({t}_{i})=\sqrt{{\sigma }_{{E}_{{\rm{on}}}}^{2}({t}_{i})-{\sigma }_{{E}_{{\rm{off}}}}^{2}({t}_{i})}\mathrm{}.$$

From these quantities the radiation power in each slice can be determined. When using the energy loss method, the radiation power in each slice is^1,2,17^

12 $$P({t}_{i})=\Delta E({t}_{i})\cdot \frac{I({t}_{i})}{e}\mathrm{}.$$

When using the energy spread method, the radiation power in each slice is^1,17,21^

13 $$P({t}_{i})\propto {\sigma }_{E}{({t}_{i})}^{2}\cdot I{({t}_{i})}^{\frac{2}{3}}.$$

To determine the total radiation power an additional, independent measurement of the total pulse energy is necessary. This can for example be accomplished using a calibrated gas detector^22–24^.

To see if the algorithm reconstructs the photon pulse correctly both the power profile obtained from the energy loss and the energy spread method are used as target power profiles for the reconstruction algorithm. By then comparing the reconstructed temporal power profiles one can observe the similarities and differences to check if even though the inputs might be slightly different the algorithm ends up with the same temporal power profile.

In the following, the photon pulses obtained from the reconstruction using only the TDS and the corresponding reconstruction methods are displayed as dashed-dotted lines. The reconstructions using the energy loss method are plotted in black and those using the energy spread method are plotted in blue. 50 different initial guesses serve as starting points for the reconstruction algorithm. These 50 reconstructions are averaged to obtain the final reconstructed photon pulses (solid lines). The mean reconstruction is surrounded by a dark gray and a light gray shaded area which is one standard deviation for the energy loss and the energy spread method, respectively. For the reconstruction the width of the Gaussian base functions *σ*_*j*_ was chosen to be 0.1 fs as well as their spacing $\Delta t={t}_{j+1}-{t}_{j}$.

### Reconstruction of photon pulses from bunches with 30 pC charge

We first apply the reconstruction algorithm the experimental data with a charge of 30 pC at the undulator entrance. The parameter overview can be found in the left column of Table 1.

**Figure 5:**
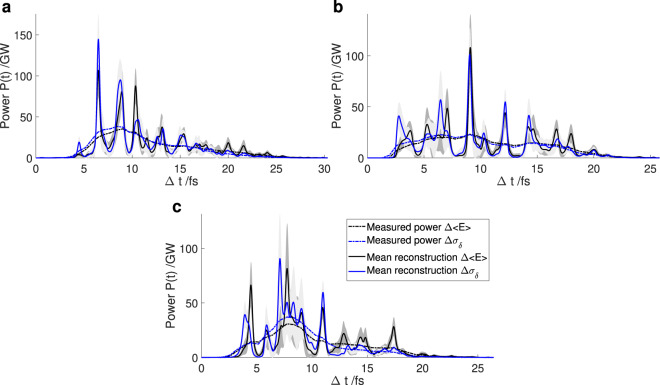
Reconstruction of a photon pulses measured at LCLS obtained using the iterative reconstruction algorithm. The total bunch charge at the undulator is 30 pC, the TDS resolution is 1.2 fs. (**a**) to (**c**) show the reconstruction of different, individual pulses.

Examples of reconstructed photon pulses can be found in Fig. 5, demonstrating the capabilities and the limitations of the algorithm when applied to measured data. As can be seen in these figures, the photon pulse obtained from the energy loss and the energy spread method differ slightly.

For the power profiles in Fig. 5a the position of most of the larger SASE spikes is the same for both reconstructions. The maximum power of the highest spike is ~1.36 times higher if the energy spread method is used as the target profile. This is because the power obtained from the TDS reconstruction at the position of the spike is ~1.30 times higher if the energy spread method is used. The smaller SASE spikes agree very well in position and also in height. The reconstruction works well, as the main SASE spikes are separated, which according to the testing using simulations facilitates the reconstruction for the algorithm.

The position and height of most of the main SASE spikes is also very similar for both target profiles shown in Fig. 5b. The maximum power of the main spike differs by less than 7%. There is a slight difference in the reconstruction on the left side of the main spike. Here, multiple spikes are very close to one another and the reconstruction algorithm reaches its limitations providing slightly different results for the two target profiles. It can be observed that the power profile contains two spikes between 2 fs and 4.5 fs and three between 4.5 fs and 8 fs. The position can be reconstructed, yet the exact maximum power for each single spike remains unknown. The isolated spikes in the region of 10 fs and 20 fs can be retrieved efficiently by the algorithm.

Figure 5c shows a reconstruction where the algorithm reaches the limitations for reconstructing the main features of the photon pulse. These limitations were noted in the testing section. In the central part of the photon pulse (between 6 fs and 10 fs) the SASE spikes are too close to one another to be retrieved by the algorithm. Nonetheless, the smaller, isolated side peaks to the left and right of the central part are retrieved adequately by the algorithm.

### Reconstruction of photon pulses from bunches with 20 pC charge

Secondly, bunches with a charge of 20 pC at the undulator entrance are used for the reconstruction. The settings are the same as in the previous section, but the bunches are truncated even further using energy collimators in the first bunch compressor. The resulting parameters can be found in the right column of Table 1.

**Figure 6:**
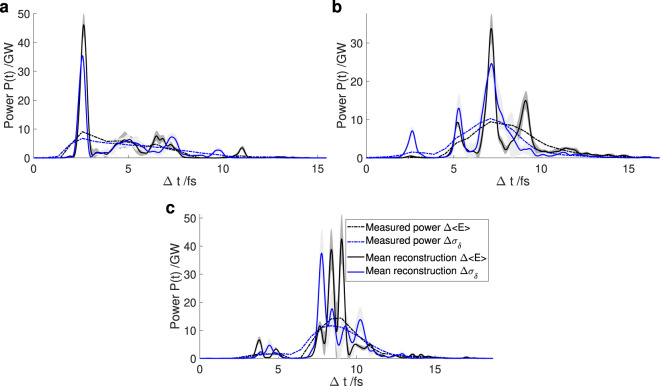
Reconstruction of photon pulses measured at LCLS obtained using the iterative reconstruction algorithm. The total bunch charge at the undulator is 20 pC, the TDS resolution is 1.0 fs. (**a**) to (**c**) show the reconstruction of different, individual pulses.

Figure 6 shows examples of reconstructed photon pulses for these settings demonstrating the capabilities and the limitations of the algorithm when applied to measured data.

The reconstruction found in Fig. 6a shows a dominant spike at the beginning of the photon pulse. Both methods reconstruct the spike at 2 fs but with a different maximum power. With the energy difference method the power is ~1.35 times higher, in good agreement with the blurred power, which is ~1.34 times higher. The rest of the photon pulse consists of smaller, dense spikes which can only partly be retrieved by the algorithm. In the region between 8 fs and 10 fs the photon pulse power reconstructed using the energy difference method is close to zero. As a result the algorithm does not reconstruct any power in that region. On the contrary, the energy spread method yields power in this region which results in the reconstruction algorithm showing spikes here. Hence, the difference in the reconstruction is not caused by instability in the algorithm, but rather by the difference in the two TDS analysis methods.

The second example for this setting can be found in Fig. 6b. The main spikes between 6 fs and 8 fs and 4 fs and 6 fs of the photon pulse are retrieved using both methods as starting points. The small spike between 2 fs and 3 fs of the photon pulse is higher if the energy spread method is used, as the photon pulse power reconstructed using this method is also higher. Several adjacent spikes in the region of 8 fs to 10 fs cannot be clearly distinguished by the reconstruction algorithm. Additionally, the power obtained by the energy difference method is higher in this region, leading to a higher power retrieved by the algorithm.

An example where the iterative algorithm did not reconstruct successfully can be found in Fig. 6c. Here, the central part of the photon pulse comprises many adjacent SASE spikes that cannot be distinguished by the algorithm. This is expected since the initial reconstruction using the two methods differs in the region of 7 fs to 11 fs both in height and shape and thus, the algorithm ends up with different solutions in this region.

## Conclusions

In this paper, the reconstruction algorithm previously published^5^ was improved and applied to real measurement data taken at LCLS.

The results show how the reconstruction algorithm using both TDS and spectral information can improve the X-ray pulse profile reconstruction over the established method using only the TDS measurement. The reconstruction accuracy is currently limited by the achievable resolution of the TDS implemented at LCLS. For future upgrades an even better accuracy can be expected. The algorithm excels at reconstructing single, isolated spikes. Multiple, adjacent spikes of similar height are more difficult to be retrieved individually.

## Data Availability

The algorithm and datasets generated during and/or analysed during the current study are available from the corresponding author on reasonable request.
